# Dataset on photocatalytic degradation of Levofloxacin using hydroxyapatite photocatalyst: Optimization by response surface methodology

**DOI:** 10.1016/j.dib.2022.108219

**Published:** 2022-04-29

**Authors:** Adrian D. Go, Francis M. dela Rosa, Drexel H. Camacho, Eric R. Punzalan

**Affiliations:** Department of Chemistry, De La Salle University, 2401 Taft Avenue, Manila 0922, Philippines

**Keywords:** Box-Behnken design, Levofloxacin (LEVO), Hydroxyapatite, Photocatalysis, Response surface methodology, Mathematical model

## Abstract

The upsurge of antibiotic usage in the 20th century has resulted in increasing levels of pharmaceutical compounds in bodies of water. A particular antibiotic, levofloxacin, is a third-generation quinolone known to target Gram-positive organisms like atypical pathogens. Chronic toxic effects of levofloxacin to some microorganisms lead to the disruption of marine ecosystems. Unfortunately, a relatively low concentration of levofloxacin in water bodies discourages researchers from exploring potential risk assessment and removal in wastewater treatment plants. In this article, aqueous levofloxacin was degraded using hydroxyapatite catalyst under UV-irradiation. Response Surface Methodology (Box Behnken Model) was used to model and optimize the degradation efficiency parameter. The response was fitted into a 2-factor interaction equation revealing a satisfactory ANOVA evaluation (R^2^=97.08%, adjusted R^2^= 94.89, predicted R^2^=91.1%). An optimal photodegradation efficiency was determined to attain the following conditions: 1.5 g/L catalyst dose, 4 ppm levofloxacin, and a pH level of 10. The model predicted a value of 71.6% degradation efficiency, which is very close to 70.6% generated experimentally.

## Specifications Table

**Table:** 

Subject	Environmental Science
Specific subject area	Photocatalysis
Type of data	Tables and Figures
How the data were acquired	Photoreactor system: Photodegradation of levofloxacin was performed in a custom reactor box (dimensions: 75 × 29 × 38 cm). Germicidal lamp (Sankyo Denki, G20T10, 20W, 588.5mm length, 32.5mm diameter, λ = 254) was positioned horizontally above the reactor. Aqueous levofloxacin solution was placed 12cm below the lamp. Levofloxacin solution was held under dark for 30 minutes prior to UV light exposure at constant stirring. Aliquots were obtained every time interval. Temperature was controlled at 20°C by continuous water flow thru the jacketed beaker containing the levofloxacin solution. UV-Vis: The spectra for the degradation were obtained using Shimadzu UV-Visible spectrophotometer UV-2600. Settings were set to 2 nm slit width, medium scan speed, and wavelength range of 200-400nm. Baseline correction using deionized water was employed before sample runs.
Data format	Raw and Analyzed
Description of data collection	Hydroxyapatite catalyst was prepared from mussel shell pre-cursor. Photocatalytic activity of hydroxyapatite was evaluated by the absorbance of levofloxacin obtained after UV irradiation. Box Behnken Modelling was used to optimize three parameters: catalyst dose (0.5-1.6 g/L), LEVO concentration (4-10 ppm), and pH (4-10). Photodegradation efficiency was obtained after 5-hour irradiation subsequently after subjecting to dark conditions (30 mins). Verification of the model was simulated using optimum conditions from the model (1.5 g/L catalyst dose, 4 ppm LEVO concentration, pH 10). An aliquot of 5 mL was obtained every 60 mins. Degradation behavior and extent were evaluated via UV-Vis spectroscopy. MINITAB 17 statistical software was used to generate the response surface model.
Data source location	Central Instrumentation Facility-De La Salle University Laguna, Biñan, Laguna, Philippines
Data accessibility	Go, Adrian; dela Rosa, Francis; Camacho, Drexel; PUNZALAN, ERIC (2022), “Dataset on photocatalytic degradation of Levofloxacin using hydroxyapatite photocatalyst: Optimization by response surface methodology”, Mendeley Data, V2, doi: 10.17632/h82gspgkpf.2 Mendeley Data repository link: https://data.mendeley.com/datasets/h82gspgkpf/2

## Value of the Data

• This work presents empirical and statistical data on the photodegradation of levofloxacin via photocatalytic process. The data given here as well as the experimental parameters exhibit the effectivity of hydroxyapatite as an alternative green photocatalyst. Researchers may opt to modify this photocatalyst to further enhance the degradation properties.
• The results of the Box-Behnken modelling and analysis can be used by other investigators to test and compare the influence of experimental parameters (catalyst dosage, LEVO concentration, and pH) on the efficiency of the system in the photodegradation of other related organic compounds.
• The data presented herein can be used in the optimization of similar photocatalytic systems (i.e. hydroxyapatite /UV) for both small and large scale studies. These data can be referenced by academic investigators looking at photocatalytic system designs, testing, and optimization. Scientific professionals working in industries such as wastewater treatment and organic waste disposal facilities may use these data in improving the current methods.

## 1 Data Description

**Fig. 1:**
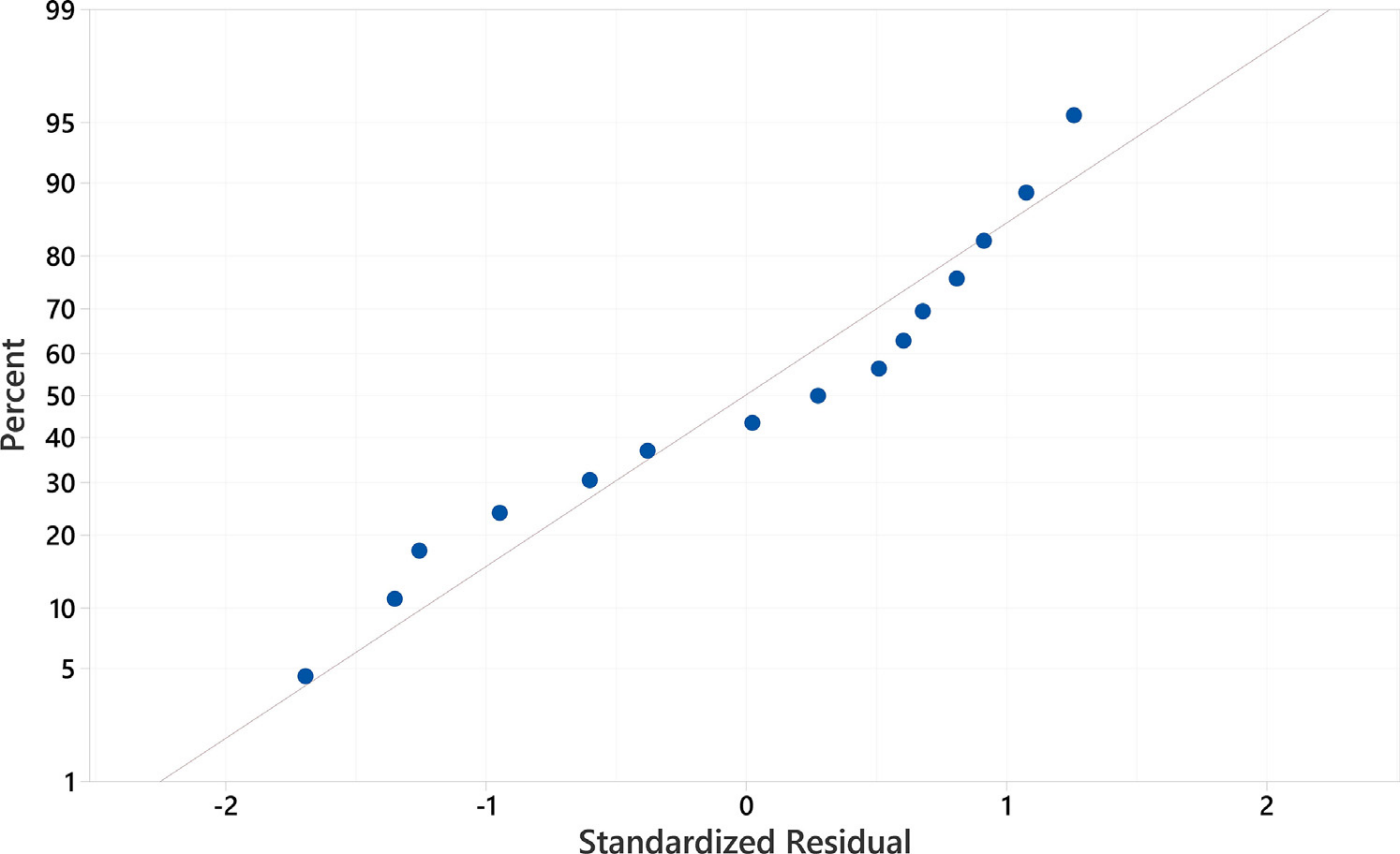
Normal probability plot of standardized residuals.

**Fig. 2:**
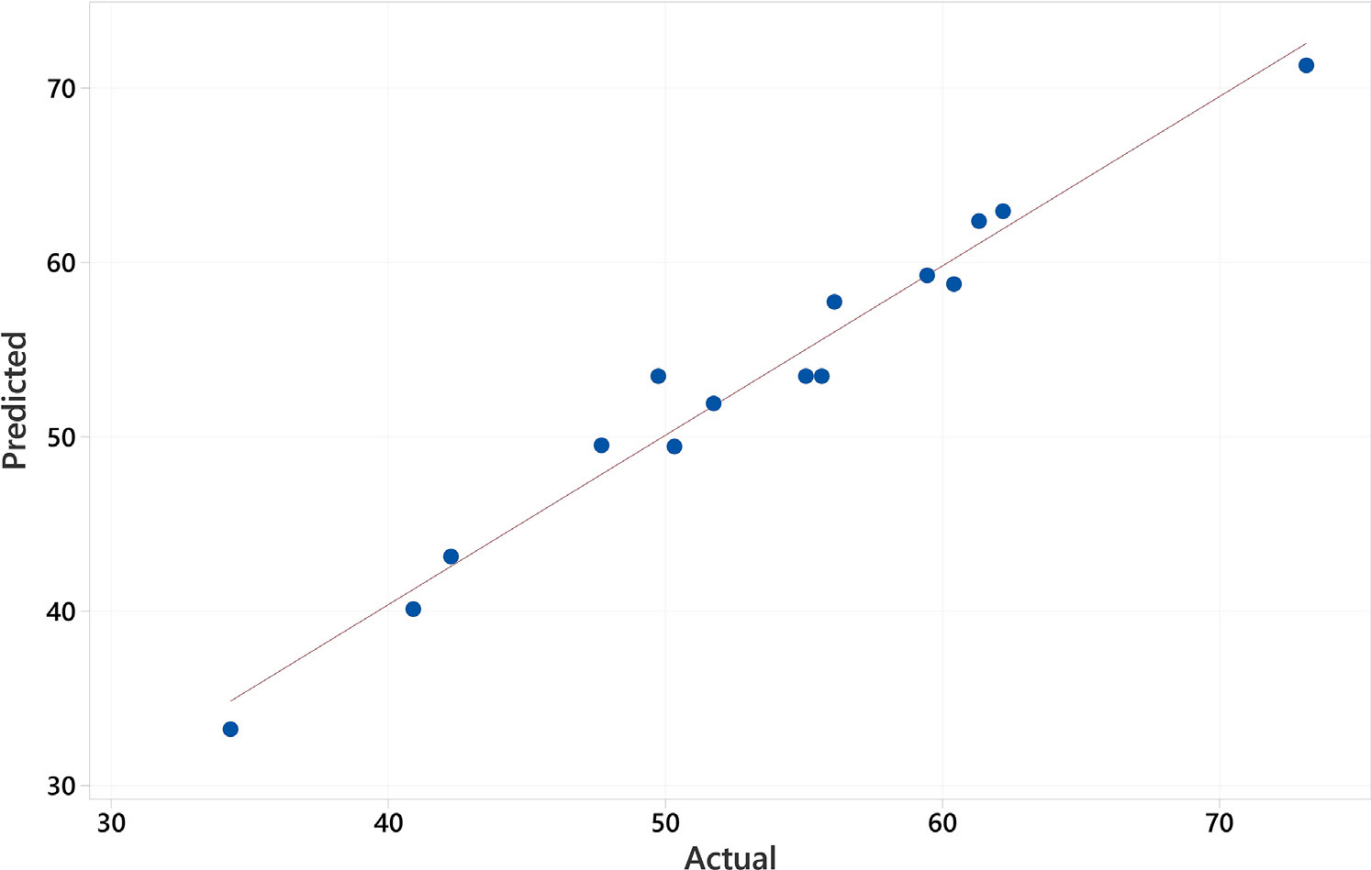
Predicted vs actual efficiency of levofloxacin photodegradation.

The range and levels of the defined variables considered in the Box Behnken design model is shown in Table 1. These are the catalyst dose (A), levofloxacin concentration (B), and pH value (C). The model equation computing the efficiency is given in Equation (1). In the model, A, B, C, AB, and AC represent the catalyst dosage, levofloxacin concentration, pH value, the interaction of catalyst dosage-levofloxacin, and the interaction of catalyst dosage-pH heavily influencing the behavior of the degradation efficiency, respectively. In Table 2, the efficiency response with different values of the three variables are enumerated. It shows photodegradation efficiency towards levofloxacin. Evaluation of the best fitted equation is given in Table 3 demonstrating that the 2-Factor Interaction describes the curve due to having the highest regression values. The analysis of variance is used to determine the significant factors that affect the photodegradation efficiency. It is also used to evaluate the quality of fit and adequacy of the model (Table 4). The Fisher (F) and probability (P) values are used to determine the significant variables. Values of "Prob > F" less than 0.0500 indicate model terms are significant whereas the opposite indicate non-significance [1]. The generated regression equation representing the photodegradation efficiency of levofloxacin is given in Equation 1. The coefficients of determination (R^2^) and adjusted R^2^ are satisfactory to support the significance of the model [2]. Normal probability plot of standardized residuals and predicted vs actual plot is shown in Fig. 1 and 2, respectively. Surface interaction plots of the variables are shown inFig. 4. Results show favorability for high catalyst dosage and pH with low levofloxacin concentration. The software response optimization was set to have the maximum response which obtained the optimum conditions of 1.5 g/L catalyst dose, 4 ppm levofloxacin concentration and pH 10. This generated a predicted efficiency of 71.6% (5 hours). The optimum conditions were simulated experimentally (Fig. 4a) and the observed photodegradation efficiency was 70.6%. Thus, verifying the efficacy of the model. The kinetics of degradation follows pseudo first order equation (Fig. 4b).

(1) Efficiency=+53.35+7.02A−7.54B+3.87C+5.23AB+7.06AC+2.03BC

**Table 1:** Range and Levels of Box Behnken Design.

Variable	low	center	high
	-1	0	+1
**(A)** Catalyst dose (g/L)	0.5	1.0	1.5
**(B)** levofloxacin concentration (ppm)	4	7	10
**(C)** pH	4	7	10

**Table 2.** Box Behnken Model Response Table.

Std. Order	(A) catalyst dose	(B) LEVO concentration	(C) pH	Efficiency	Predicted Efficiency
**1**	0.5	4	7	60.41	58.77
**2**	1.5	4	7	61.31	62.37
**3**	0.5	10	7	34.30	33.24
**4**	1.5	10	7	56.10	57.74
**5**	0.5	7	4	47.69	49.52
**6**	1.5	7	4	50.32	49.45
**7**	0.5	7	10	42.26	43.14
**8**	1.5	7	10	73.13	71.31
**9**	1.0	4	4	59.44	59.26
**10**	1.0	10	4	40.90	40.13
**11**	1.0	4	10	62.18	62.95
**12**	1.0	10	10	51.74	51.92
**13**	1.0	7	7	49.74	53.48
**14**	1.0	7	7	55.64	53.48
**15**	1.0	7	7	55.07	53.48

**Table 3.** Evaluated equation form.

Model	Lack of fit (P-value)	R^2^	Adjusted R^2^	Predicted R^2^
Linear	0.237	72.70%	65.26%	43.12%
2-Factor Interaction	0.906	97.08%	94.89%	91.11%
Quadratic	0.702	97.13%	91.97%	75.94%

**Table 4.** ANOVA results for the model.

Source	DF	Adj SS	Adj MS	F-Value	P-Value	Remarks
Model	6	1294.11	215.685	44.36	0.000	significant
Linear	3	969.13	323.044	66.44	0.000	significant
(A) catalyst dosage	1	394.8	394.805	81.20	0.000	significant
(B)LEVO conc	1	454.51	454.511	93.48	0.000	significant
(C)pH	1	119.82	119.815	24.64	0.001	significant
2-Way Interaction	3	324.98	108.326	22.28	0.000	significant
(AB)catalyst dosage*LEVO conc	1	109.2	109.203	22.46	0.001	significant
(BC)catalyst dosage*pH	1	199.37	199.374	41.00	0.000	significant
(AC) LEVO dosage*pH	1	16.4	16.402	3.37	0.104	not significant
Error	8	38.9	4.862			
Lack-of-Fit	6	17.72	2.953	0.28	0.906	not significant
Pure Error	2	21.18	10.591			
Total	14	1333.01				
SS	2.7659					
R^2^	97.08%					
Adjusted R^2^	94.89%					
Predicted R^2^	91.11%

**Fig. 3:**
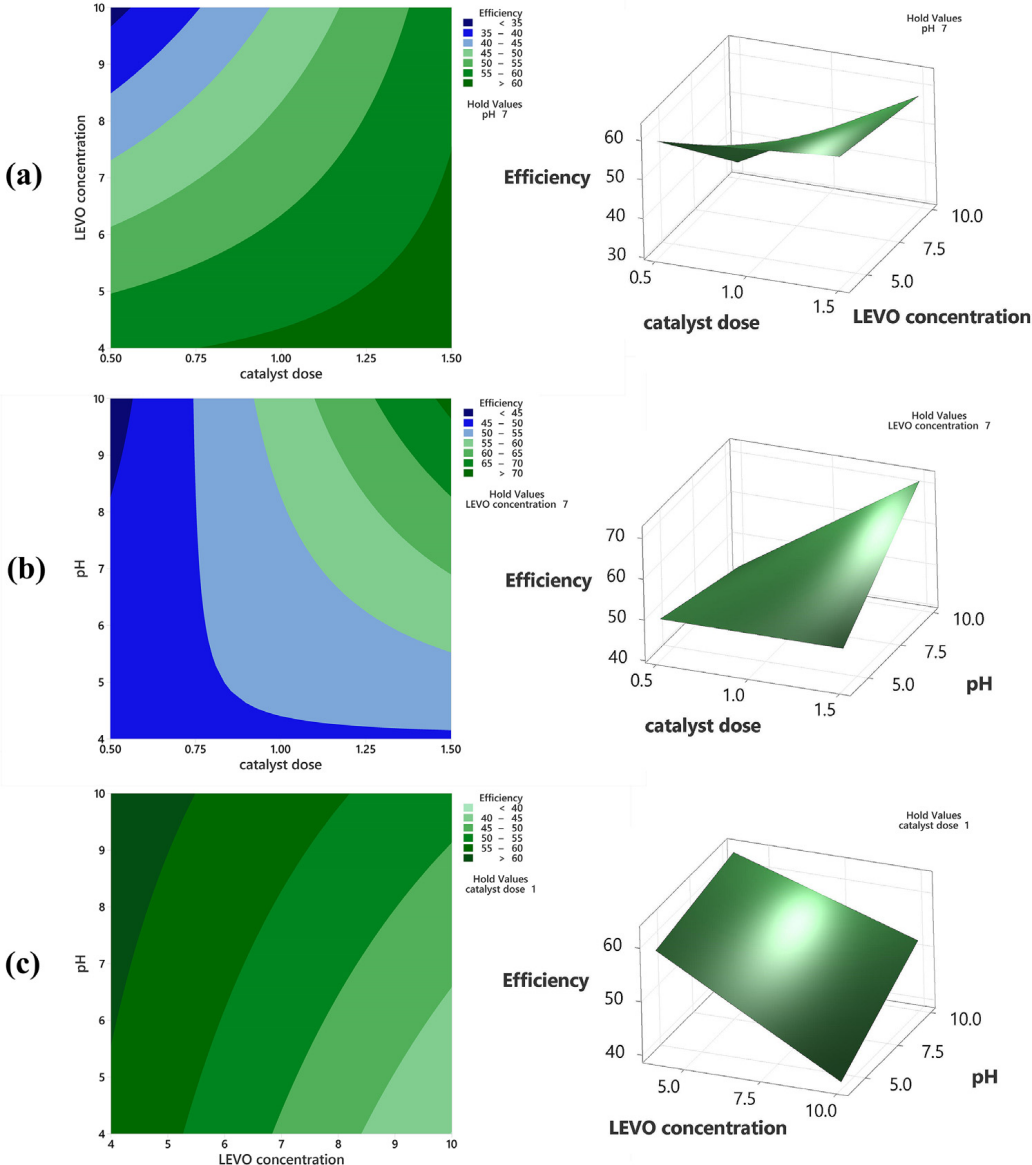
Contour and surface plots of levofloxacin photodegradation efficiency.

**Fig. 4:**
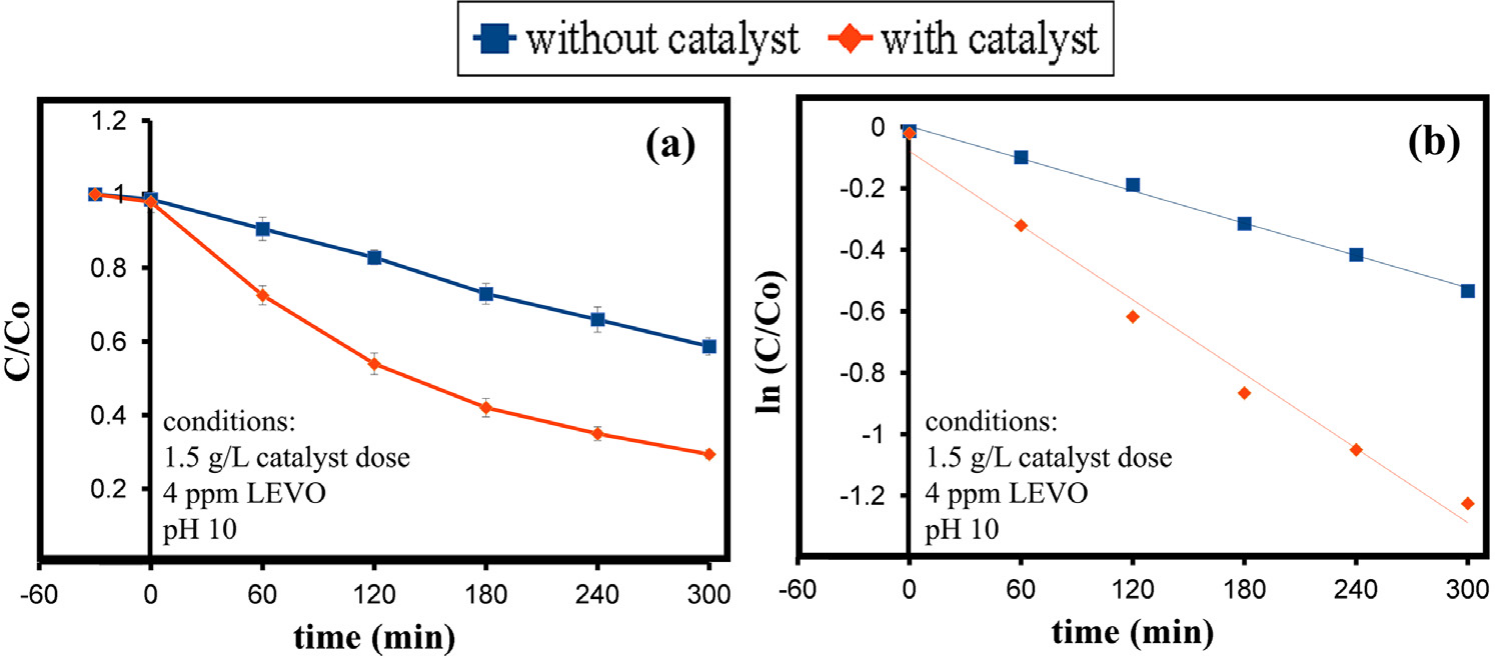
a) Photodegradation of levofloxacin (5 hours), b) kinetics of the photodegradation.

## 2 Experimental Design, Materials and Methods

### 2.1 Materials

The materials used for the study were Levofloxacin (C_18_H_20_FN_3_O_4_) (>98%, HPLC, Sigma Aldrich), nitric acid (Emsure), ammonium hydroxide (Qualikelms), ammonium dihydrogen orthophosphate (Ajax Finechem). Mussel shells (*Perna virdis*) were purchased from a local wet market.

### 2.2 Hydroxyapatite preparation

Biowaste mussel shells were soaked and cleaned with boiling water and were left to dry. The dried shells were crushed using mortar and pestle and calcined at 1000°C using a furnace. Powdered calcium oxide was produced after this step and was finely grounded using mortar and pestle. Grounded CaO weighing 1.00g was acquired and dissolved in a 20 mL of 2.50 M HNO_3_ forming Ca(NO_3_)_2_ solution. The pH of this solution was then adjusted to 10 using ammonium hydroxide. To this solution, 25 mL of 0.427 M (NH_4_)_2_HPO_4_ was added dropwise under sonication (40 Hz) for 1 hour. The resulting mixture was centrifuged, washed with deionized water, and dried overnight at 100^°^C to obtain the hydroxyapatite powder [3].

### 2.3 Determination of levofloxacin photodegradation efficiency

The photodegradation of Levofloxacin was carried out in a reactor that was designed and built in our laboratory. The type of UV lamp used was Sankyo Denki 20 W, λ = 254 monochromatic light. A constant flow of water (20°C) in between the jacketed beaker was set to control the temperature. A mass of Hydroxyapatite was transferred to a 100 mL levofloxacin solution with constant stirring. The pH of the reaction mixture was adjusted by careful addition of a solution of NaOH or HCl. The solution was allowed to stand while stirring in the dark for 30 mins and then was UV-illuminated at constant stirring (400 RPM). The acquired sample solution was centrifuged to remove the suspension. The extent of degradation was determined spectrophotometrically using Shimadzu UV-Visible spectrophotometer UV-2600 at 287 nm. Percent degradation efficiency was calculated as follows [4,5],

(2) $$\%\text{Degradation}\;\text{Efficiency}\;=\frac{Co-C}{Co}x100\%$$

Where Co = initial concentration and C= variable concentration

### 2.4 Experimental design

#### 2.4.1 Box-Behnken experiments

The software used for the modeling is Minitab 17. The model is based on Box-Behnken design with 15 experimental runs. The parameters analyzed were (A) catalyst dose, (B) LEVO concentration, and (C) pH. Photodegradation efficiency (after 5 -hour UV-irradiation) was set as the response. Predicted optimum parameters for the photodegradation were obtained after setting the response to maximum.

#### 2.4.2 Verification of Box-Behnken model

Predicted parameters (1.5 g/L catalyst dose, 4ppm LEVO, and pH 10) from the model were simulated in an actual experiment. An aliquot (5mL) of the levofloxacin solution was obtained every 60-minute interval to determine the concentration and evaluate the photodegradation behavior.

## Ethics Statements

N/A

## CRediT Author Statement

**Adrian Go:** Investigation, Methodology, Writing – original draft; **Francis dela Rosa:** Formal analysis, Writing – review & editing; **Drexel Camacho:** Resources, Writing – review & editing; **Eric Punzalan:** Supervision, Visualization, Writing – review & editing.

## Declaration of Competing Interest

The authors declare that they have no known competing financial interests or personal relationships that could have appeared to influence the work reported in this paper.

## Data Availability

Dataset on photocatalytic degradation of Levofloxacin using hydroxyapatite photocatalyst: Optimization by response surface methodology (Original data) (Mendeley Data).
